# *In vitro* methods for evaluating therapeutic ultrasound exposures: present-day models and future innovations

**DOI:** 10.1186/2050-5736-1-21

**Published:** 2013-11-01

**Authors:** Ahmad Alassaf, Adham Aleid, Victor Frenkel

**Affiliations:** 1 Department of Biomedical Engineering, Catholic University of America, 620 Michigan Ave NE, Washington, DC 20064, USA

**Keywords:** Therapeutic ultrasound, Ultrasound bioeffects, *In vitro* methods, *Ex vivo* tissues, Tissue-mimicking phantoms, Biological scaffolds

## Abstract

Although preclinical experiments are ultimately required to evaluate new therapeutic ultrasound exposures and devices prior to clinical trials, *in vitro* experiments can play an important role in the developmental process. A variety of *in vitro* methods have been developed, where each of these has demonstrated their utility for various test purposes. These include inert tissue-mimicking phantoms, which can incorporate thermocouples or cells and *ex vivo* tissue. Cell-based methods have also been used, both in monolayer and suspension. More biologically relevant platforms have also shown utility, such as blood clots and collagen gels. Each of these methods possesses characteristics that are well suited for various well-defined investigative goals. None, however, incorporate all the properties of real tissues, which include a 3D environment and live cells that may be maintained long-term post-treatment. This review is intended to provide an overview of the existing application-specific *in vitro* methods available to therapeutic ultrasound investigators, highlighting their advantages and limitations. Additional reporting is presented on the exciting and emerging field of 3D biological scaffolds, employing methods and materials adapted from tissue engineering. This type of platform holds much promise for achieving more representative conditions of those found *in vivo*, especially important for the newest sphere of therapeutic applications, based on molecular changes that may be generated in response to non-destructive exposures.

## Introduction

It was more than 60 years ago when therapeutic ultrasound (TUS) exposures were first shown to be beneficial in medical practice. In a seminal preclinical study, continuous, low energy, and non-focused exposures were shown to stimulate the formation of bone callus in a radial fracture model in rabbits [1]. Since then, interest and development in the field of TUS has continued to grow, where presently hundreds of research centers and universities worldwide are working to develop and improve applications in the fields of vascular disease, oncology, and physical therapy [2]. Whereas non-focused, low intensity TUS exposures are being used in the clinic for healing [3] and to enhance local transdermal delivery [4], focused ultrasound (FUS) is being employed for thermally ablating uterine fibroids [5] and a variety of malignant tumors including those in the prostate [6], breast [7], pancreas [8], and bone [9]. As FUS becomes more accepted, additional solid tumors (e.g., in the kidney and liver) will similarly be routinely treated on an outpatient basis [10].

Although *in vivo* preclinical studies are ultimately required to evaluate new TUS devices and procedures prior to clinical trials, it is always desirable, when possible, to carry out studies *in vitro* in order to minimize animal experimentation, lower costs and variability, and increase throughput. In this review, a summary of the existing *in vitro* methods will be provided, detailing the manner by which each method is appropriate for a specific investigational purpose. This will be preceded by a short section on some conventional *ex vivo* methods that are commonly used. Finally, a section will be presented on a new *in vitro* platform presently in development, based on 2D and 3D biological scaffold models of soft tissues.

### *Ex vivo* tissues

Although not considered a true *in vitro* method, using *ex vivo* tissue is similar to other *in vitro* methods in that it is used in lieu of carrying out the exposures *in vivo*. One of the most common uses of *ex vivo* tissue is for evaluating new and experimental FUS devices for thermally ablating tissue. The literature is replete with such studies, where the purpose is to visualize lesion formation, temperature elevations, or both. *Ex vivo* tissues that have been used for this purpose include turkey breast [11], canine prostate [12], bovine muscle [13], and porcine kidney [14]. These tissue models can be useful for initial tests for creating lesions in predictable locations. However, because of the lack of perfusion (and subsequent convective heat loss), lesion formation will occur at relative lower rates of energy deposition, where this must be taken into account for dosimetry-based studies.

### *In vitro* methods

#### Tissue-mimicking materials

**Figure 1:**
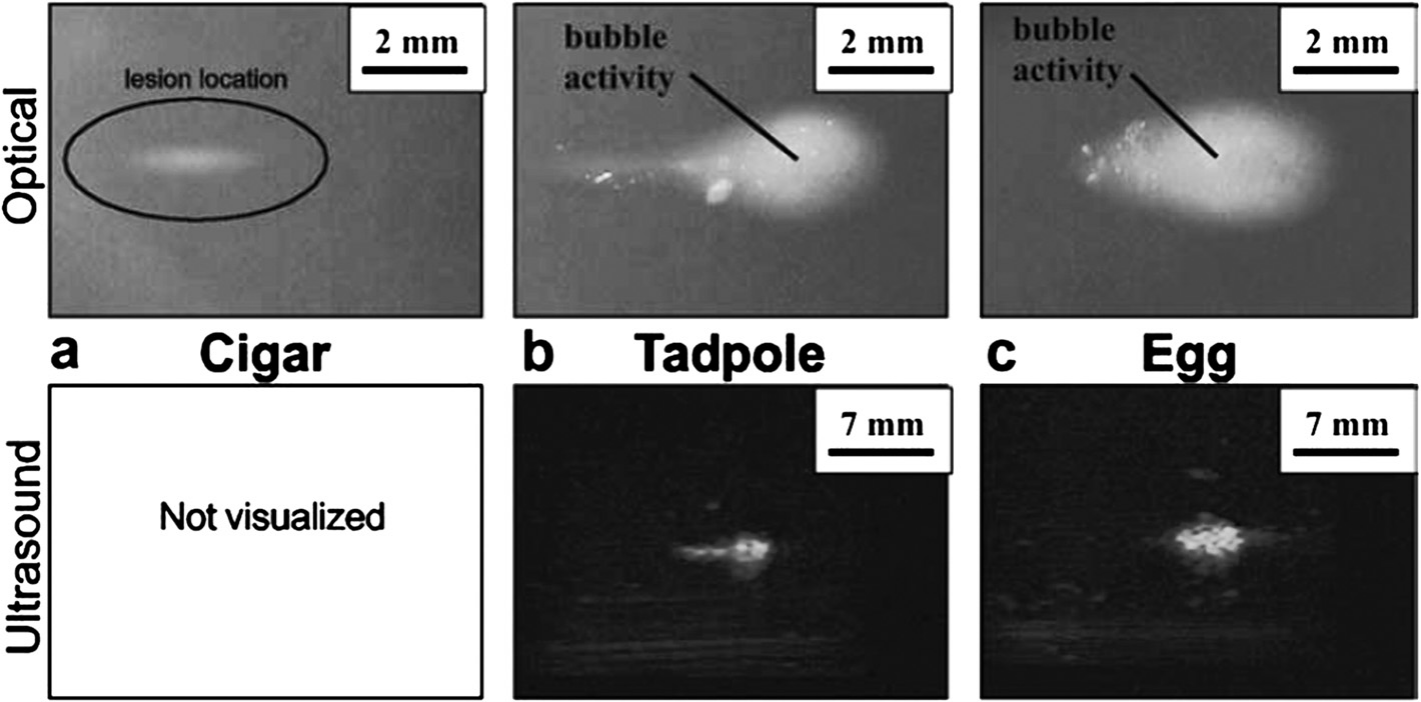
**Optical and ultrasound visualization of different types of lesions in 6% BSA polyacrylamide TMM phantoms.** The 'cigar’-shaped lesions **(a)** are typically created through thermal mechanisms only. The 'tadpole’-shaped **(b)** and 'egg’-shaped **(c)** lesions on the other hand are created by acoustic cavitation activity in the prefocal region (the FUS ultrasound transducer was on the right side). This interpretation is supported by the fact that the cavitation-based lesions are more visible by ultrasound due to the enhanced echogenicity of these regions (reprinted with permission from [11]).

Perhaps the most widely used *in vitro* method for testing FUS exposures are phantoms made from tissue-mimicking materials (TMMs) such as polyacrylamide hydrogels [11,15]. The phantoms are translucent, allowing thermal lesions to be visualized optically, in addition to being detectable with diagnostic ultrasound. Bovine serum albumin (BSA) is also added to these phantoms as a heat-sensitive protein and to increase the attenuation coefficient of the TMM. When heated sufficiently, the BSA denatures, creating the visible lesion. These phantoms can also be produced in any shape or size, depending on the container in which they are made. One disadvantage of these TMMs is that even when using relatively high concentrations of BSA, the attenuation coefficient is still well below that of normal tissue (where the attenuation coefficient is the most important tissue characteristic for the generation of heat [16]). Therefore, relatively greater levels of energy will be required to produce a thermal lesion when compared to a typical soft tissue [11]. Another disadvantage is that the formation of the lesions is an irreversible process; hence, the phantoms cannot be reused. The manner by which these phantoms can be employed is demonstrated in Figure 1.

Recently, more advanced TMMs have been developed, possessing characteristics of soft tissue important for investigating the thermal effects of FUS exposures. One such TMM was produced from gellan gum, a high-temperature hydrogel matrix, which was combined with various-sized aluminum oxide particles and other constituents. This TMM was shown to be reusable when generating temperature elevations sufficient for thermal ablation of the tissue [17]. In a follow-up study, thermocouples were embedded in the TMM, demonstrating its utility for characterizing temperature elevations generated with these exposures [18].

### Tissue-based methods

A variety of *in vitro* TUS studies have been carried out using what can be termed 'tissue-based’ methods. One of the most popular are blood clots made from fresh, whole blood confined in an acoustically compatible material. In one such study, 1 ml of whole blood was collected from healthy volunteers and closed off in appropriately sized sections of pediatric Penrose tubing. Pulsed FUS (pFUS) exposures followed by immersions in tissue plasminogen activator (tPA) were subsequently shown to improve thrombolysis when compared to the tPA on its own [19]. This same *in vitro* clot model was used in a follow-up study to help elucidate the manner by which these enhanced therapeutic results were obtained. Investigations showed improved bioavailability of the tPA in the clots, where the methods used included scanning electron microscopy, fluorescently tagged antibodies specific to the tPA, and fluorescence recovery after photobleaching [20]. In another *in vitro* clot study, the tPA was radiolabeled with ^125^I. Using a gamma counter on serial sections of the clots, this study showed how low energy, non-focused ultrasound (LEnFUS) exposures could improve the penetration of the agent into the clots [21].

Other *in vitro* methods may also be considered tissue-based even though they do not contain any original components of tissue. They are, however, comprised of one or more purified components found in tissue, where the structural function of these molecules realistically represents those found *in vivo*. One example is the use of fibrin gels, fibrin being an insoluble protein produced in response to bleeding. It is a major component of a blood clot, arranged in long fibrous chains. Its structural function is to entangle platelets, leading to the formation of a clot. A number of studies have been carried out with purified fibrin gels, using LEnFUS. The exposures were shown to create a number of effects important for enhancing thrombolysis. These included structurally induced changes for enhancing flow through the fibrin [22], as well as other changes for improving binding of tPA to the fibrin itself, a requirement for fibrinolysis [23].

**Figure 2:**
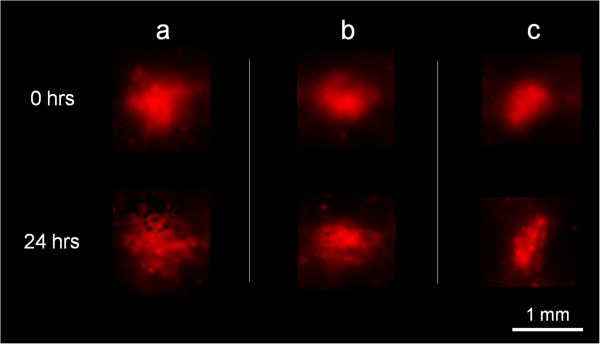
**Nanoparticle uptake in type I collagen gels.** pFUS exposures in the gels were provided at a single location, after which the gels were immersed in a suspension of 100-nm diameter, fluorescently labeled, polystyrene NPs. In all three gels **(a**, **b**, and **c)**, the NPs were initially taken up only in the region of treatment. Even at 24 h later, the NPs were somewhat more diffuse but still found to be restricted to the treated region (VF, unpublished).

Collagen is another naturally occurring polymer in the body, whose structure can also affect the delivery of drugs [2]. Fibrillar collagen in the extracellular matrix of solid tumors, for example, can limit interstitial transport, preventing sufficient and uniform delivery of anticancer agents. This is especially true in the case of large agents such as viral gene delivery vectors whose size can be greater than the spaces between the fibers [24,25]. Studies on transport have been carried out in collagen type 1 gels, looking at permeability, diffusion, and convection for tracer molecules [26]. Similar collagen gels (the collagen being the same type found in the extracellular matrix of mammalian tissue) were used to investigate the effect of pFUS exposures on transport. The exposures were previously shown to generate gaps between parenchymal cells in animal models of both skeletal muscle [27] and solid tumors [28]. These structural changes increased the effective pore size of the tissue, resulting in enhanced convective mass transport of injected nanoparticles (NPs). The gels were given similar exposures and then immersed in the same fluorescently labeled NPs, 100 nm in diameter. Macroscopic fluorescent imaging showed the particles to initially be taken up only in the region of the focal zone. Twenty-four hours later, the NPs were still in the same region, where they were also shown to diffuse freely in the same gels without collagen (Figure 2). Similar to the effects reported previously in solid tumors [28], skeletal muscle [27], and blood clots [19,20], it is thought that the repetitive radiation force-induced displacements produced by the exposures may have created structural alterations; specifically the disruption of the organizational structure of the collagen fibers. As in the other studies, these effects could have potentially enabled improved transport through the gels (VF, unpublished).

### Cultured cells

Studies on the effects of ultrasound are being performed with cultured cells [29,30], using either adherent cells in monolayer [31] or cells in suspension [32]. The appeal of having a controlled and reproducible medium of living cells is understandably attractive, which can facilitate high-throughput experimentation at relatively low cost when compared to animal studies. These experimental setups are, however, problematic from a variety of perspectives. The cells in suspension are of course not representative of *in vivo* conditions. Ultrasound exposures can generate streaming in the fluid as a result of the attenuation of energy [33]. This can induce mixing of the cells, creating conditions that are even further from those found *in vivo*. The same goes for a single layer of cells in culture wells, where the majority *in vitro* ultrasound studies are done. Here, the cells are backed on one side by incompressible plastic and on the other by a comparatively large volume of unconfined fluid. Furthermore, ultrasound transmission through culture wells is inefficient, resulting in mode conversion, heat generation, and the potential formation of standing waves within the cell volume. These factors combined can lead to uncertainties of up to 700% in the actual ultrasound exposure experienced by the cells [30].

**Figure 3:**
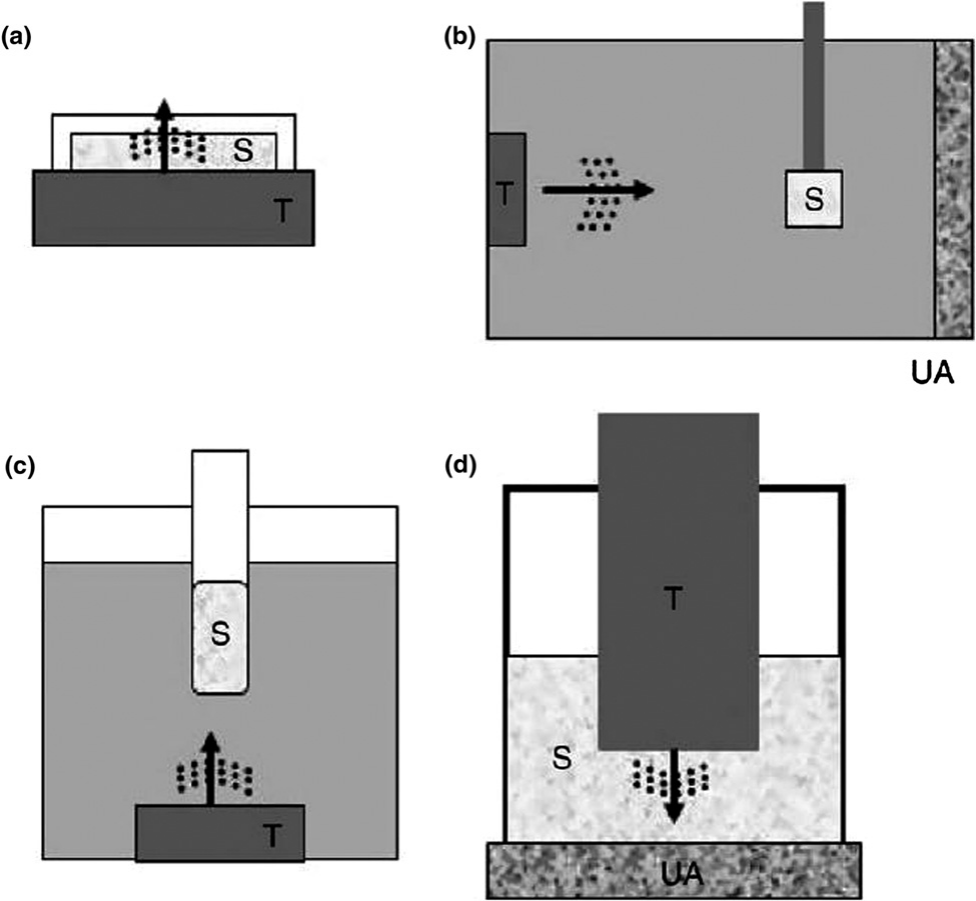
**Experimental setups for ultrasound treatment of cultured cells. (a)** The ultrasound transducer (T) is positioned directly below a culture well containing the cells (S). Acoustic gel is used to couple between the transducer and the well. **(b)** Degassed water is used to couple between the transducer and the sample. An ultrasound absorber (UA) is used to prevent the reflection of the ultrasound waves. **(c)** Similar to setup B however with a variation in orientation. **(d)** The ultrasound transducer is inserted into the well. This setup is typically used for small samples in 24- or 96-well plates (reprinted with permission from [29]).

A comparatively large number of *in vitro* studies have been carried out investigating sonoporation (i.e., the use of ultrasound to generate pores to enhance drug and gene delivery to individual cells [34]). These include studies, for example, that use ultrasound contrast agents to enhance acoustic cavitation for this purpose [35]. These studies, typically carried out in a monolayer of cells, in open culture wells, typify the lack of suitability of these experimental setups for representing *in vivo* conditions. As will be described later in this review, one of the most important factors controlling cavitation activity is the geometry in which the bubble is confined [36]. The different possible experimental configurations for *in vitro* studies with cultured cells appear in Figure 3.

### Non-biological-based methods

**Figure 4:**
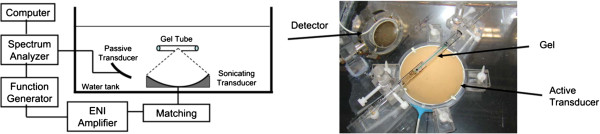
**Experimental setup for investigating cavitation activity in agar gel tunnels.** (left) A schematic representation of the setup showing the integration of the different elements that were used. (right) A photograph of the setup showing the gel, the FUS transducer, and the cavitation detector. All the components were in an acrylic tank filled with degassed water used for coupling. Microbubbles were injected into the tunnels just prior to the exposures (reprinted with permission from [36]).

So far, all the *in vitro* methods that have been discussed have involved attempts to reproduce, to one degree or another, the *in vivo* environment, where one or more biological components are included. Systems, however, have also been developed to investigate the effects of only a single and very specific characteristic, where actual biological components were not required. One example is the work of Sassaroli and Hynynen who carried out extensive investigations into the manner by which the diameter of a vessel will affect various aspects of acoustic cavitation activity, including the resonance frequency and the damping coefficient [36-38]. The importance of these studies was based on the principle that bubble activity under the geometrical confines of a blood vessel can be very different than that in free field (i.e., in an unconfined or infinite medium). In addition to mathematical modeling and simulations, the investigators also developed a number of experimental setups. These involved a FUS transducer directed at micron-sized tubes, at which a passive cavitation detector was also directed. Among the factors that were investigated was the relationship between the diameter of the tube and the acoustic pressure threshold for the induction of cavitation. Earlier studies used tubes made from silica and polyester [38]. More recently, they extended their investigations to using agar gels, in which tunnels were created to more realistically simulate small blood vessels *in vivo*[36]. This experimental setup appears in Figure 4.

### Biological scaffolds

Living tissues are exposed to a multiplicity of internal and external environments, influencing their growth and regeneration. One of the major design factors in tissue engineering is creating *in vitro* environments comparable to native tissue for growing cells and tissues [39]. The surroundings of living cells in the body include a three-dimensional (3D) architecture, where interactions occur between cells, as well as between individual cells and the extracellular matrix (ECM). Despite this, the vast majority of *in vitro* studies are carried out in 2D cultures for the sake of simplicity. This type of environment will ultimately lead to the development of cells that are physiologically compromised [40]. 3D scaffolds, for example, were found to be superior to 2D cultures for neural cell differentiation from embryonic stem cells [41]. For many studies, such as testing cells for sensitivity to drugs, 2D cultures may be sufficient [42]. However, it is widely accepted that 3D scaffolds are essential for realistically evaluating the effects of mechanical stimuli on cells, especially in terms of both their morphology and biochemical responses through the process of mechanotransduction [43]. These mechanical stimuli can include dynamic compression [44], intermittent hydrostatic pressure [45], rotating shear stress [46], and ultrasound [47]. In addition to 3D organization, clinically relevant cell biology research using *in vitro* models requires the multicellular complexity of an organ, as well as an ECM for the required cell interactivity, while still allowing a variety of experimental interventions to be performed [40].

3D biological scaffolds have shown great potential in applications in regenerative medicine, such as for healing of bone fractures [48]. In addition to providing physical support for the cells, these structures may provide a variety of functions including the modulation of signaling pathways for growth, proliferation and differentiation of the cells, as well as for their survival [49]. 3D biological scaffolds may also serve as a reproducible platform for a host of biological investigations. A number of studies have been carried out using TUS and 3D biological scaffolds. In one, chondrocytes were seeded in chitosan scaffolds and exposed to LEnFUS. Compared to controls, the cells in the treated scaffolds had higher cellular viability and higher levels of type II collagen in the extracellular matrix [47]. In another study, pulsed LEnFUS was shown to increase adhesion of osteoblast precursor cells in trabecular calcium phosphate scaffolds [50]. Pretreating scaffolds to ultrasound prior to adding cells may also be beneficial. A study in decellularized patella tendon scaffolds, for example, demonstrated that using pulsed LEnFUS at relatively higher intensities could produce a more porous matrix without adversely affecting the biochemical constituents or damaging the architecture of the scaffolds. The microscopic alterations were shown to improve penetration and subsequently, recellularization of primary tenocytes [51]. These studies were performed to help elucidate the underlying mechanisms involved when using ultrasound in physical therapy for regenerative purposes. In each study, however, both the type of scaffold and the cells were different, as were the experimental setups and ultrasound devices. To date, there are no standardized platforms available to ultrasound investigators that can realistically reproduce conditions *in vivo* in a consistent and cost-effective manner; especially for soft tissue models.

In our laboratory, we are presently developing scaffolds specifically designed for evaluating the biological effects of TUS exposures. The methodology for preparing these scaffolds is based on existing ones for bone-mimicking scaffolds, typically incorporating naturally occurring polymers such as gelatin and collagen [52]. Our scaffolds possess characteristics of soft tissues, being comprised of chitosan and gelatin. Whereas chitosan confers beneficial structural characteristics to the scaffolds [53], gelatin contains favorable cell-binding properties [54]. This formulation, for example, is being used to develop implantable dermal constructs to which an epidermal layer would then be adhered [55]. One of the attractive features of the 3D scaffolds is that they can be formed into any shape or size, determined by the container (i.e., mold) in which they are produced. A deeper and narrower design, for example, could be more suitable for a focused beam. A broader and shallower scaffold on the other hand could be used for exposures provided with planar, non-focused transducers.

**Figure 5:**
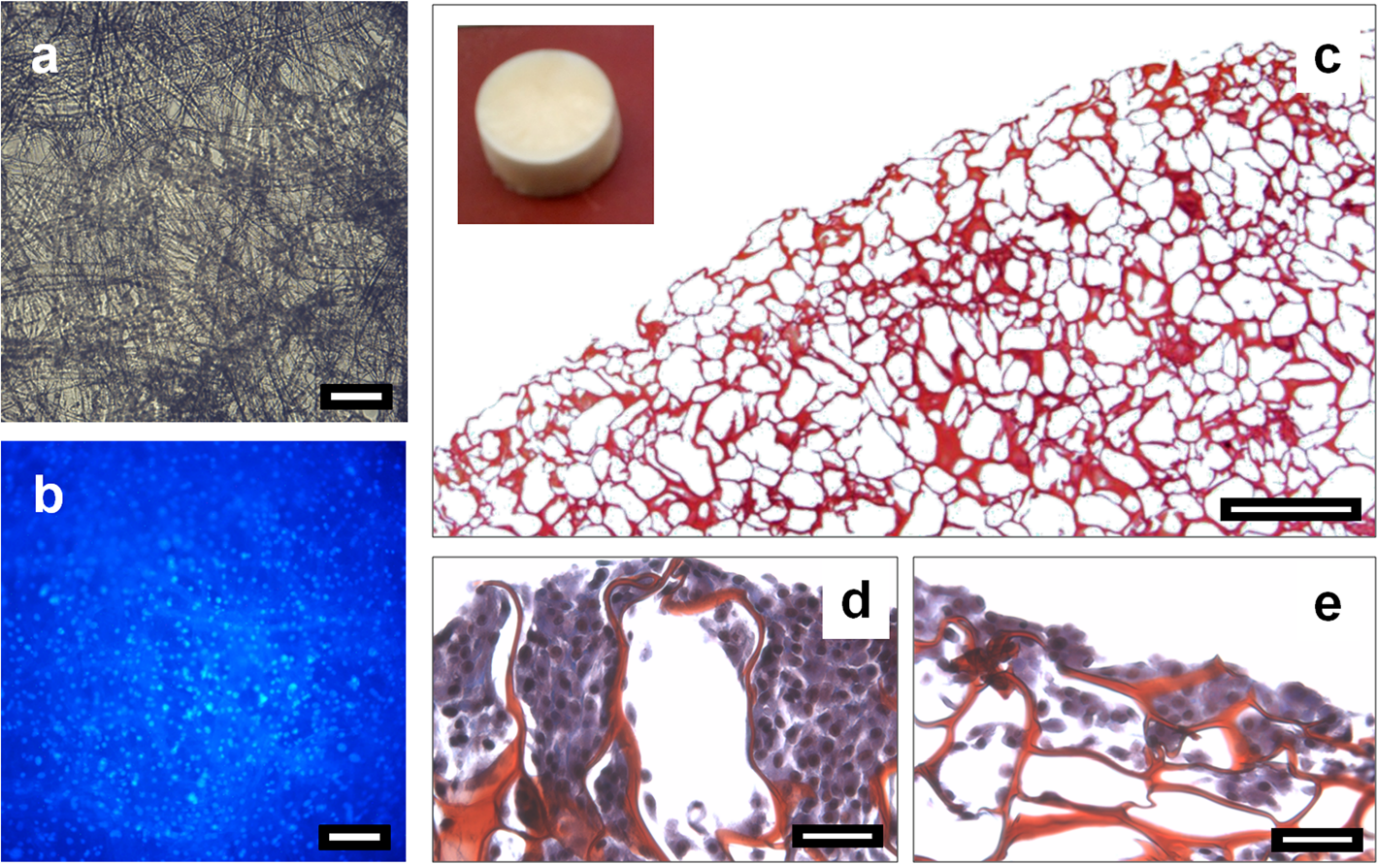
**Chitosan-gelatin biological scaffolds.** (left) 2D scaffold: **(a)** brightfield image showing the fibrous structure of the scaffold; **(b)** fluorescent image of the same scaffold in **(a)**, where the nuclei of fibroblasts are visible, stained with DAPI. Bar = 100 μm. (right) 3D scaffolds sectioned, stained with Masson's trichrome (red, scaffold; purple, fibroblasts), and observed with brightfield microscopy. **(c)** Edge region of a non-cellularized scaffold (bar = 200 μm). (inset) Entire scaffold (height = 7 mm; radius = 20 mm). **(d**,**e)** Regions of cellularized scaffolds (outer surface at top) (bar = 50 μm). Pore sizes range from 50 to 200 μm, with various degrees of cellularization.

One of the exciting possibilities that we have begun investigating is the use of 2D scaffolds 'rolled up’ in to pseudo-blood vessels that would then be embedded in an inert gel phantom, which would provide structural support for the vessel. A blood-mimicking fluid [56] could then be circulated through the vessel while ultrasound exposures are being carried out. Such a setup would allow investigations of ultrasound-mediated drug delivery applications. This includes sonoporation [57], and also the deployment of drugs from temperature sensitive liposomes [58]. Examples of both 2D and 3D biological scaffolds that we have been preparing appear in Figure 5.

In addition to those already discussed, there are other advantages of the proposed 3D scaffolds over the other *in vitro* platforms described so far in regard to the investigational methodologies that they could potentially facilitate. One is that essentially, any cell type could be used. This includes cells that are stably transfected with reporter genes whose signals, fluorescent (e.g., green fluorescent protein) or bioluminescent (e.g., firefly luciferase), could be imaged *in situ*. Using promoters for specific genes to be investigated, such as the gene for heat shock proteins that respond to the generation of heat [59], repeated imaging session could be carried out for temporal characterization of expression over a protracted period post-treatment. Other methodologies that could be employed, and which would not be possible *in vivo*, include *in situ* fixation [60], for 'capturing’ discreet and transient structural alterations occurring during the exposures, and *in situ* hybridization [61], for looking at spatial patterns of gene expression in individual cells. The ability to accurately correlate structural alterations with induced patterns of gene expression would enable investigations into phenomena such as mechanotransduction, the mechanism by which mechanical signals are converted by cells into biochemical responses [62].

## Conclusions

With the advancement of TUS has come a large and impressive variety of *in vitro* methods and platforms for evaluating these exposures and the devices being developed to apply them (Table 1). Some have been simple and straightforward, such as encasing whole blood to represent an acute blood clot. Others have been more sophisticated, as for tissue-mimicking phantoms fabricated from a combination of materials through a complex process, which can also incorporate thermocouples for characterizing induced temperature elevations. Each one of these methods has been innovative and effectively served the specific purpose of the tests being carried out. They also have contributed to reducing the requirement on animal testing, in addition to reducing variability and costs, and expediting the evaluation process.

**Table 1:** **A comparison of the different *in vitro *** **methods**

**Method**	**3D environment**	**Acoustic compatibility**	**Live cells**
*Ex vivo* tissue	**+**	**+**	**-**
Tissue-mimicking materials	**+**	**+**	**-**
Tissue-based methods	**+**	**+**	**-**
Cultured cells	**-**	**-**	**+**
Non-biological methods	**+**	**+/-**	**-**
Biological scaffolds	**+**	**+**	**+**

Today, biological scaffolds are being developed to evaluate TUS exposures, incorporating live cells in a 3D environment. These will be used specifically for evaluating the molecular effects that the exposures can generate and contribute to the investigative process for determining the potential of applications based on these effects. As new applications of TUS continue to be proposed and developed, one would expect that novel *in vitro* test methods and platforms will also arise, offering investigators an even richer and diverse range of options to facilitate the process, as well as reduce the demand on animal testing.

## Competing interests

The authors declare that they have no competing interests.

## Authors’ contributions

VF was responsible for conceptualizing the outline of the manuscript. All authors contributed equally to collecting the referenced studies and their organization into the text. All authors read and approved the final manuscript.
